# Impact of daily artificial gravity on autonomic cardiovascular control following 60-day head-down tilt bed rest

**DOI:** 10.3389/fcvm.2023.1250727

**Published:** 2023-10-23

**Authors:** J.-N. Hoenemann, S. Moestl, A. Diedrich, E. Mulder, T. Frett, G. Petrat, W. Pustowalow, M. Arz, M.-T. Schmitz, K. Heusser, S. M. C. Lee, J. Jordan, J. Tank, F. Hoffmann

**Affiliations:** ^1^ Institute of Aerospace Medicine, German Aerospace Center, Cologne, Germany; ^2^ Department of Internal Medicine III, Division of Cardiology, Pneumology, Angiology, and Intensive Care, University of Cologne, Cologne, Germany; ^3^ Department of Medicine, Division of Clinical Pharmacology, Autonomic Dysfunction Service, Vanderbilt University, Nashville, TN, United States; ^4^ Institute of Medical Biometry, Informatics and Epidemiology (IMBIE), University Hospital Bonn, Bonn, Germany; ^5^ Wyle Laboratories, Life Sciences and Systems Division, Houston, TX, United States; ^6^ Head of Aerospace Medicine, University of Cologne, Germany, Cologne

**Keywords:** bed rest, artificial gravity, cardiovascular deconditioning, autonomic nervous system, tilt table test

## Abstract

Impaired cardiovascular autonomic control following space flight or immobilization may limit the ability to cope with additional hemodynamic stimuli. Head-down tilt bedrest is an established terrestrial analog for space flight and offers the opportunity to test potential countermeasures for autonomic cardiovascular deconditioning. Previous studies revealed a possible benefit of daily artificial gravity on cardiovascular autonomic control following head-down tilt bedrest, but there is a need for efficiency in a long-term study before an artificial gravity facility would be brought to space. We hypothesized that artificial gravity through short-arm centrifugation attenuates functional adaptions of autonomic function during head-down tilt bed rest. 24 healthy persons (8 women, 33.4 ± 9.3 years, 24.3 ± 2.1 kg/m^2^) participated in the 60-day head-down tilt bed rest (AGBRESA) study. They were assigned to three groups, 30 min/day continuous, or 6(5 min intermittent short-arm centrifugation, or a control group. We assessed autonomic cardiovascular control in the supine position and in 5 minutes 80° head-up tilt position before and immediately after bed rest. We computed heart rate variability (HRV) in the time (rmssd) and frequency domain, blood pressure variability, and baroreflex sensitivity (BRS). RR interval corrected rmssd was reduced supine (*p* = 0.0358) and during HUT (*p* = 0.0161). Heart rate variability in the high-frequency band (hf-RRI; *p* = 0.0004) and BRS (*p* < 0.0001) decreased, whereas blood pressure variability in the low-frequency band (lf-SBP, *p* = 0.0008) increased following bedrest in all groups. We did not detect significant interactions between bedrest and interventions. We conclude that up to daily 30 min of artificial gravity on a short-arm centrifuge with 1Gz at the center of mass do not suffice to prevent changes in autonomic cardiovascular control following 60-day of 6° head-down tilt bed rest.

**Clinical Trial Registration**: https://drks.de/search/en/trial/DRKS00015677, identifier, DRKS00015677

## Introduction

The autonomic nervous system regulates the cardiovascular system, which enables human beings to maintain blood pressure and organ perfusion in the face of environmental challenges including those experienced during and following spaceflight (1, 2). Indeed, hemodynamic stresses imposed by extravehicular activities in space or by standing on Earth or another celestial body could not be sustained without counter-regulatory autonomic nervous system adjustments (3). When autonomic nerves are completely disabled, the human cardiovascular systems cannot respond to gravitational stress, physical exertion (4), or other environmental challenges. Space conditions may negatively affect the autonomic nervous system (5), which could limit the resilience of the cardiovascular system. Moreover, the autonomic nervous system is engaged by weightlessness-related changes in cardiovascular structure or function, such as cardiovascular deconditioning or volume changes, which further limit the capacity to respond to environmental challenges (6).

Non-invasive measurements of heart rate variability, blood pressure variability, and baroreflex sensitivity are considered to reflect autonomic influences on the sinus node and on vascular tone and can be utilized to track changes in cardiovascular autonomic control (7–9). Such measurements could have utility in identifying astronauts with limited autonomic counterregulatory capacity, in guiding countermeasure development, and, ultimately, in individualizing countermeasure deployment (10–12). Therefore, we assessed heart rate variability, blood pressure variability, and baroreflex sensitivity in the supine position and during hemodynamic stress through head-up tilt testing before and after 60-day of six-degree head-down tilt bed rest, which is an established terrestrial spaceflight analog with or without daily artificial gravity (13). Daily artificial gravity elicited through short arm centrifugation had a beneficial effect on cardiovascular function in studies lasting from 5 to 21 days (14, 15). Given the limited number of exposes persons in these studies and the longer duration of many space missions, there is a need for additional longer-term head-down bed rest studies.

The study was part of the artificial gravity bed rest ESA study (AGBRESA, German Clinical Trials Register DRKS00015677), which tested the efficacy of daily artificial gravity through short-arm centrifugation as a multipurpose countermeasure. Hereby, we focused on adjustments in autonomic function leading to changes in the balance between sympathetic and parasympathetic cardiovascular modulation following head-down tilt bedrest. Therefore, typical changes following space flight and bed rest, namely reductions in baroreflex-mediated parasympathetic heart rate control and enhanced sympathetic modulation of the heart and the vasculature should be attenuated through daily artificial gravity (16–18). We hypothesized that cardiovascular deconditioning during bed rest would affect cardiovascular autonomic control at rest and more so during orthostatic stress and that artificial gravity attenuates these changes.

## Material and methods

### Participants

Our study is part of the NASA/ESA/DLR 60-day −6° head-down-tilt bed rest study AGBRESA, which was conducted at the DLR:envihab research facility. The methodology and study design were discussed and determined by the advisory boards of these international space agencies. Detailed criteria including a consort flow sheet regarding inclusion, exclusion, psychological, and medical screening procedures have been recently published (19). Participants who were physically and psychologically healthy, aged between 24 and 55 years, owing a body mass index between 19 and 30 kg/m², and were non-smokers were potentially eligible. Criteria for non-inclusion comprised requirement for prescription medications such as contraceptives and health conditions that would preclude participation, such as history of cardiovascular disorders including syncope, musculoskeletal, neurological, metabolic, or endocrine disorders. Women had to have a 26–32 days menstrual cycle. We enrolled 24 healthy persons (8 women/ 16 men, mean age 33.4 ± 9.3 years, mean BMI 24.3 ± 2.1 kg/m^2^; mean ± standard deviation), All subjects provided written informed consent prior to study entry. The North Rhine Medical Association Ethics Committee approved the study.

### Study design and protocol

Study design and standardization measures have been described elsewhere (19). Briefly, following a 14-day ambulatory baseline period at the DLR:envihab facility, participants underwent 60-day of head-down tilt bed rest followed by a 14-day recovery period. To exclude possible confounders, every participant was on a highly standardized diet tailored to individual resting metabolic rates with the goal to maintain body weight within 3% of that measured at first day of bed rest over the whole study phase. Daily fluid intake from food and beverages was maintained at 50 ml/kg body weight per day at baseline and during bed rest. Daily sodium intake was 1.76 ± 0.26 mmol/kg body weight/day. Throughout the study, participants were awakened at 06:30 a.m. and lights were turned off at 11:00 p.m.

**Figure 1:**
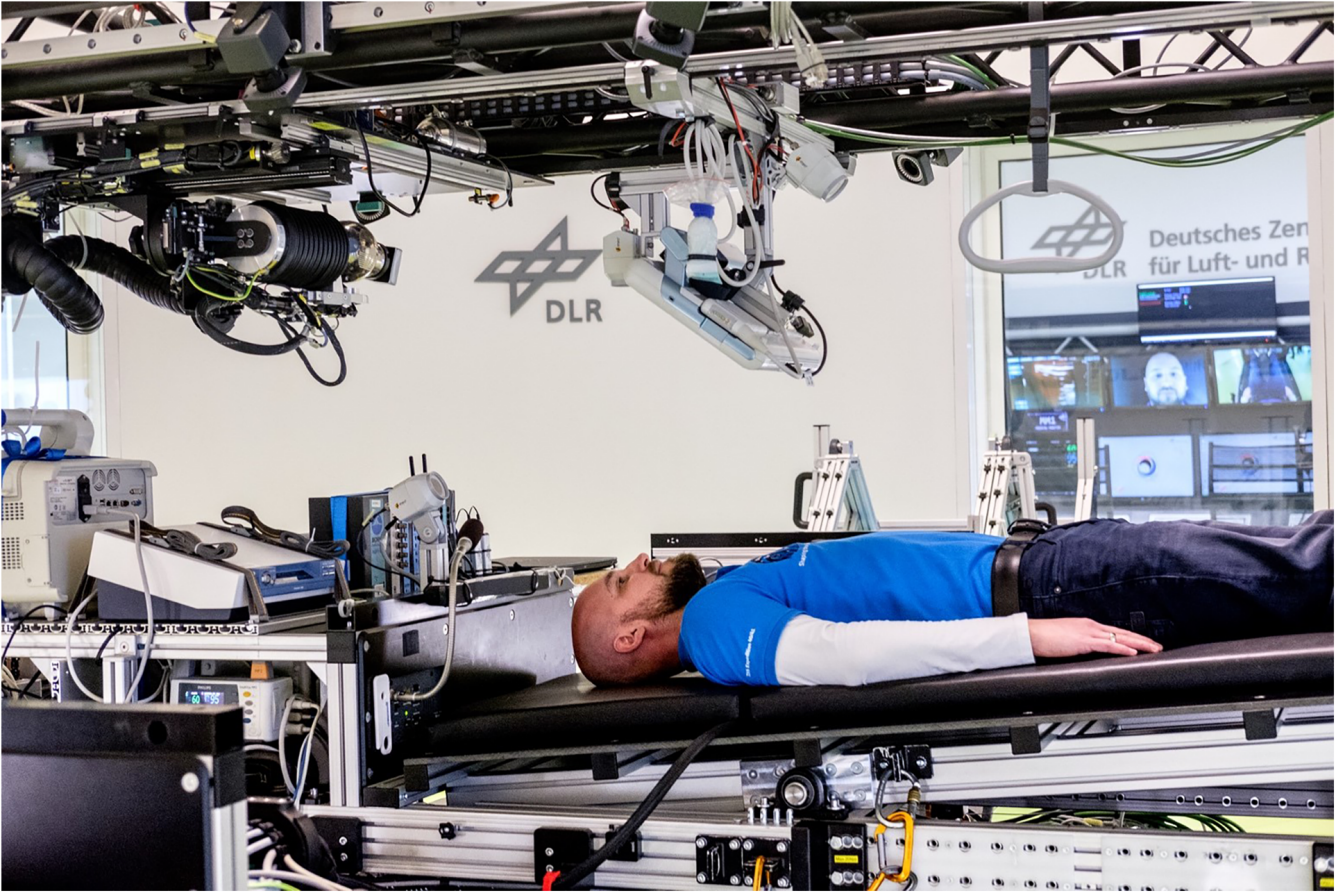
Centrifugation withn the short-arm human centrifuge was conducted in the DLR:envihab research facility. Participants were placed in the centrifuge with their heads facing the center. One Gz was to be achieved at the center of mass. An electrocardiogram with three leads, an oscillometric blood pressure cuff, finger blood pressure, and video telemetry were used in the study for safety reasons.

During the bed rest period, participants were assigned to a control group without countermeasure, or two different artificial gravity interventions. One artificial gravity group underwent 30 min continuous centrifugation. The other group underwent 6 × 5 min daily artificial gravity with 3 min breaks. Centrifugation elicited gravitational forces of 1Gz at the center of mass. For safety reasons, heart rate was measured continuously by 3-lead electrocardiogram and blood pressure was assessed intermittently with an oscillometric brachial cuff during centrifugation (Figure 1).

### Head up tilt test

**Figure 2:**
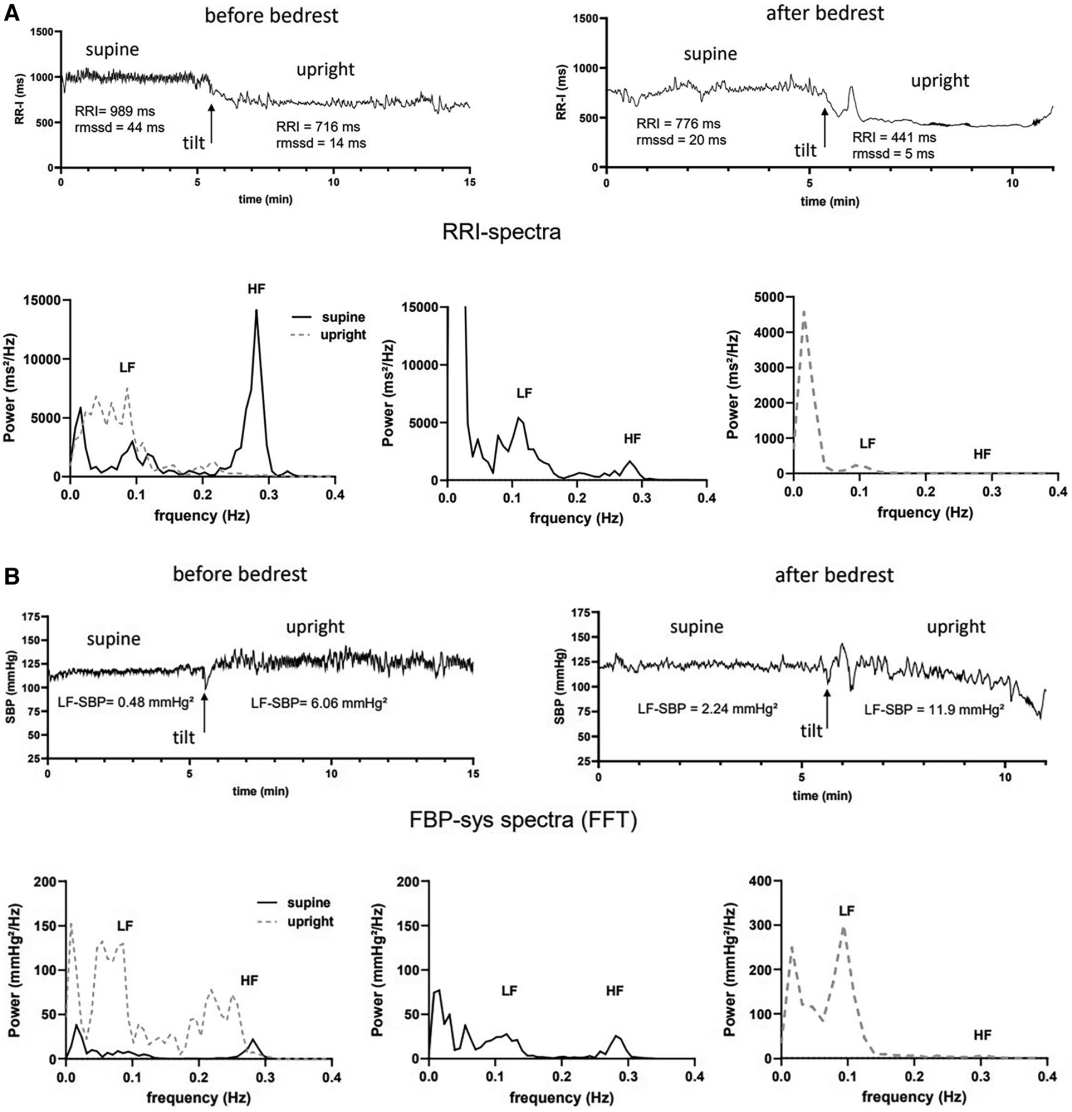
Example of heart rate and systolic blood pressure variability analysis before and after bedrest supine and during 80° head-up tilt table testing (HUT) in one subject of the intermittent artificial gravity group. (**A**) RR-intervals (RRI in ms) supine and upright early after tilting before (top left) and after bedrest (top right) are shown together with the mean heart rate (HR) and root mean squared of successive differences (rmssd) are shown. Spectral power of HRV is shown for supine (black, solid line) and upright (grey, dashed line) measurements before (bottom left) and after bedrest (bottom middle and right panel). Maximum power in the low- (lf) and high frequency range (hf) are marked. (**B**) Systolic finger blood pressure values (FBP-sys in mmHg) supine and upright early after tilting before (top left) and after bedrest (top right) are shown together with the mean values of systolic blood pressure variability in the low frequency range (lf-SBP) are shown. Spectral power of FBP-sys is shown for supine (black, solid line) and upright (grey, dashed line) measurements before (bottom left) and after bedrest (bottom middle and right panel). Maximum power in the low- (lf) and high frequency range (hf) are marked.

We conducted tilt table testing 5 days before entering bed rest and immediately following 60-day bed rest in the morning hours after a light breakfast. We recorded heart rate through a three-lead electrocardiogram and finger blood pressure (Finapres, Ohmeda Medical Instruments, Netherlands) continuously, and oscillometric brachial blood pressure in intervals of two minutes. We adjusted for body position-related hydrostatic pressure influences on finger blood pressure (Finapres corrector). Following instrumentation, which required 10–15 min, participants remained in the supine position for additional 15 min followed by 5 min baseline recordings. Then, we tilted participants up to 80°. For upright heart rate and blood pressure variability as well as baroreflex sensitivity measurements, we analyzed the first five minutes during 80° head-up tilt after excluding the initial dynamic response in the first minute based on the inspection of a trained scientist. In participants unable to stand long enough, a minimum of three minutes stable recording after the initial dynamic phase was required for spectral analysis (Figure 2A).

### Heart rate variability

Rmssd based on international standard recommendations was used to describe HRV in the time domain, which reflects respiratory sinus arrhythmia and baroreflex mediated parasympathetic heart rate control (20). Moreover, we corrected rmssd values by the mean RR-Interval to account for influences of increased supine and upright heart rates after bed rest (21). We utilized spectral analysis to assess heart rate variability considering the current guidelines on heart rate variability for short-term signal acquisition (22). Details of HRV analysis were described elsewhere (23). In short, beat-to-beat time series were interpolated and resampled at 4 Hz, and the power spectra density was estimated using the Welsh method with zero padding, linear trend elimination. and a 50% overlapped Hanning window. The five minutes intervals were analyzed using overlapped segments of 128 s (512 samples). Overlapping was used in order to reduce the Hanning window effects and the variation of the power spectral density. We calculated the low-frequency power (lf-RRI, 0.04–0.15 Hz), high-frequency power (hf-RRI, 0.15–0.4 Hz), and the lf-to-hf ratio (lf/hf). Hf-RRI is also a marker for parasympathetic HR control, while lf-RRI reflects both sympathetic and parasympathetic heart rate control. The lf/hf ratio or so called sympathovagal balance describes, which frequency band prevails in autonomic heart rate control. We used natural logarithmic values of HRV parameters for statistical analysis to reduce scatter and to reach a more normal distribution of the usually exponentially distributed HRV parameters (24).

### Baroreflex sensitivity

We calculated spontaneous baroreflex sensitivity by determining the slope of the linear regression line between systolic blood pressures and subsequent RR intervals, utilizing the sequence technique (23, 25). We analyzed sequences containing at least three intervals, with 0.5 mm Hg blood pressure changes and 5 ms RR interval changes, only if the correlation coefficients were greater than 0.85. We also determined baroreflex sensitivity using cross-spectral analysis between RR intervals and systolic blood pressure variability in the low-frequency band if the coherence in the frequency band exceeded 0.5 (26). For emphasizing vascular sympathetic efferent modulation, mean power of systolic blood pressure variability in the low frequency band was also determined (lf-SBP) (27).

### Statistical analysis

We report results as mean ± standard deviation. We logarithmically transformed HRV parameters as indicated. Based on the prospective study design and to account for few missing values, we applied linear mixed-effects models to assess changes in autonomic function following head-down tilt bed rest. For each parameter, we fitted a random intercept model with fixed effects for bed rest, body position in tilt table testing, and intervention group. Adjustments for age and sex were included. *P* < 0.05 indicated statistical significance. Respecting the explorative character of the analysis, we did adjust *p*-values for multiple tests. The data supporting our results are available from the corresponding author upon reasonable request.

## Results

### Representative heart rate and blood pressure variability responses

Representative heart rate and systolic blood pressure variability analyses before and after bedrest are shown in Figure 2. The participant was a healthy, 26-years-old women who was assigned to the intermittent artificial gravity group. Compared with baseline measurements, supine RRI had decreased by 213 ms after bedrest. After bedrest, supine HRV in the time domain after bedrest was less than half the value before bedrest. Indeed, rmssd decreased from 44 ms at baseline to 20 ms following head down tilt bedrest. Respiratory sinus arrhythmia was high at baseline before bedrest with peak spectral power in the high frequency band of about 15,000 ms²/Hz, (Figure 2A). Supine blood pressure variability (lf-SBP), which is mediated through sympathetic modulation of vascular tone, was lower before than after bedrest, (Figure 2B). The participant tolerated 17 min head-up tilt before bedrest and only 6 min of passive upright tilt following bedrest as described previously (28). The orthostatic decrease of RRI was 273 ms before and 335 ms bpm after bedrest consistent with increases in sympathetic efferent activity and post bedrest postural tachycardia. Low frequency power of HRV increased during head up tilt, which led to an increase of the lf/hf ratio indicating a shift from primarily vagal heart rate control to a dominant sympathetic heart rate control. HRV in the low and high frequency bands was almost abolished at the heart rate of 137 bpm during the first minutes of head-up tilt after bedrest. In contrast, blood pressure variability was low before bedrest, (Figure 2B). Mean and peak lf-SBP variability increased briskly with head up tilt but more so after bedrest.

### Autonomic heart rate control and baroreflex sensitivity

**Figure 3:**
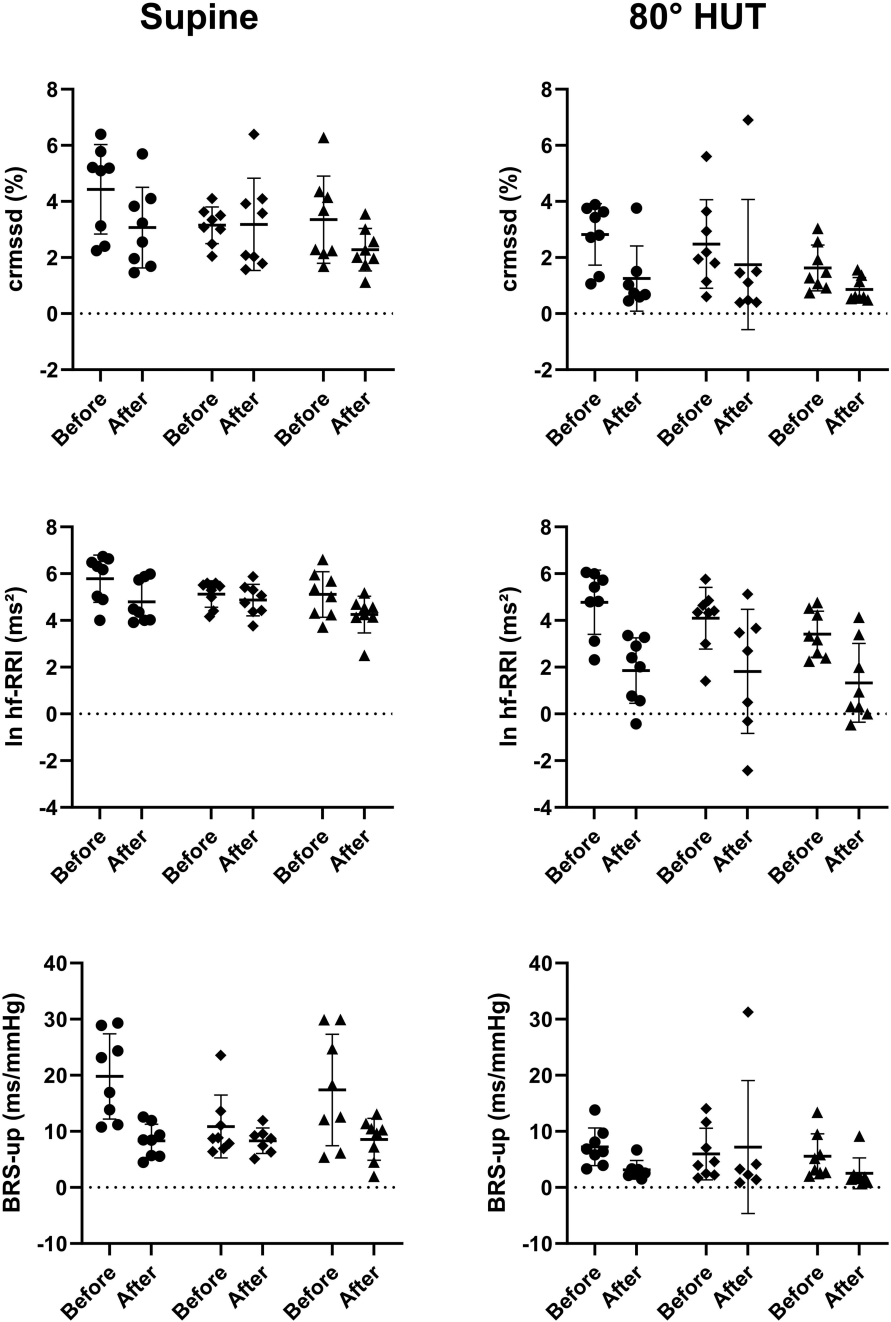
Individual values of transformed parameters of heart rate variability (crmssd = RR-interval corrected root mean square of successive differences; ln hf-RRI = logarithmic high frequency power of RR-interval variability) and baroreflex sensitivity (BRS-up = baroreflex sensitivity calculated with the sequence technique for increasing systolic blood pressure) reflecting parasympathetic heart rate control while supine (left) and during 80° head-up-tilt (HUT, right) before and after bed rest shown as scatterplots. The horizontal bars indicate the mean value plus/minus standard deviation (circle: control group, square: continuous artificial gravity group, triangle: intermittent artificial gravity group).

**Figure 4:**
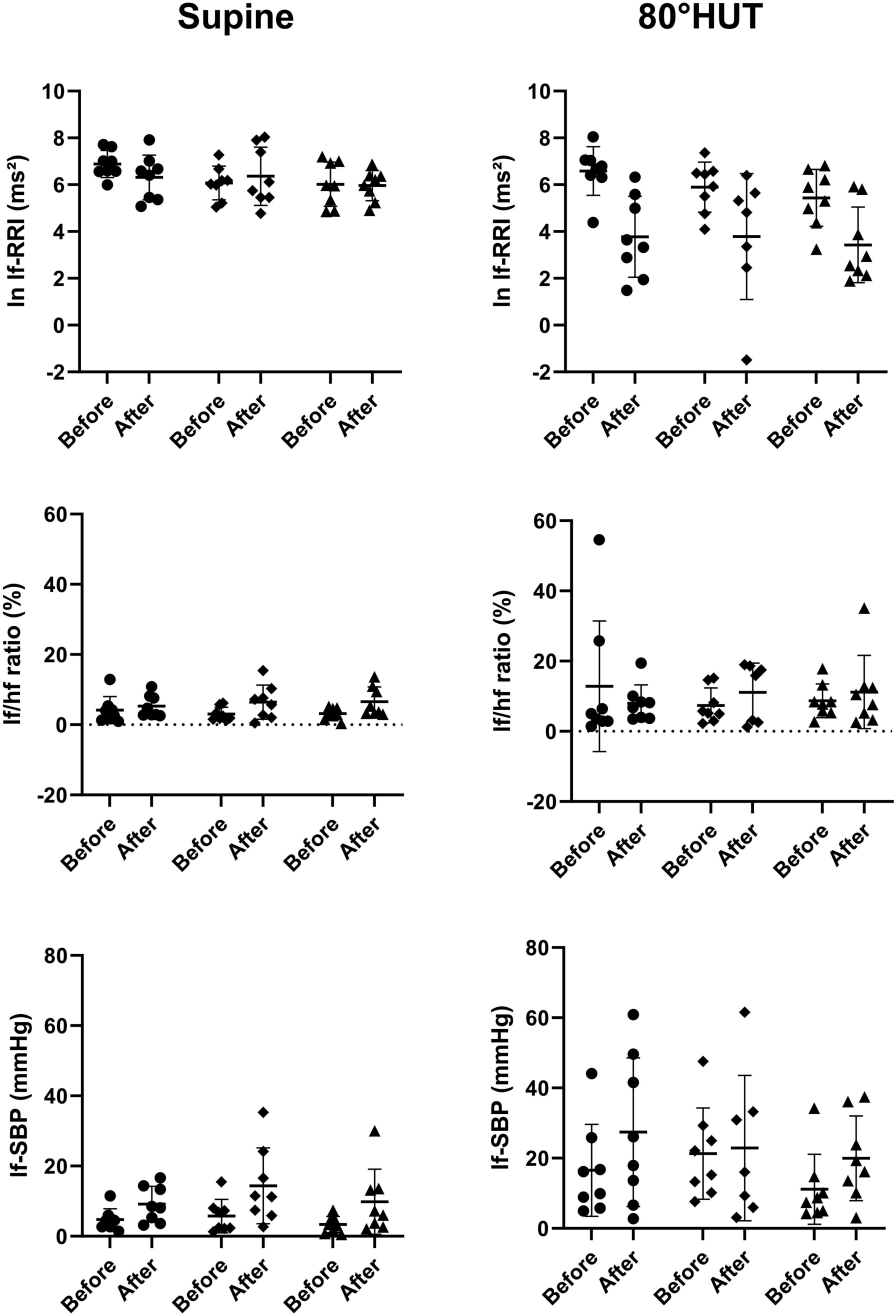
Individual values of transformed and proportional parameters heart rate- and blood pressure variability parameters in the low frequency range reflecting at least in part sympathetic heart rate control (ln lf-RRI = logarithmic low frequency power of RR-interval variability; lf/hf ratio = sympathovagal balance) and vascular efferent control (lf-SBP = systolic blood pressure variability power in the low-frequency range) while supine and during 80° head-up-tilt before and after bed rest. The horizontal bars indicate the mean value plus/minus standard deviation (circle: control group, square: continuous artificial gravity group, triangle: intermittent artificial gravity group).

The RR-interval was reduced in all groups following bed rest in supine position and showed an even steeper decrease during head up tilt (Table 4). Rmssd was significantly reduced following bed rest even after correction for the mean RR-interval (Table 3 upper row). HRV in the respiratory frequency band was significantly reduced following bed rest in supine and 80° HUT, but revealed a stronger decrease in artificial gravity groups. However, logarithmic representation (Figure 3 middle row) and normalization revealed no group differences. Baroreflex mediated heart rate control measured as reduction of heart rate following increases in blood pressure tended to be less affected in artificial gravity groups (Figure 3 lower row). The intervention groups show a larger scatter during passive standing compared to a more pronounced and homogenous drop in BRS in the control group. HRV in the low frequency range (lf-RRI) did not change significantly at baseline supine after 60-day of head-down tilt bed rest. However, lf-RRI was significantly lower upright at 80° HUT after bedrest compared to values before bedrest (Figure 4, upper row). Lf/hf ratio following 60-day head-down tilt bed rest increased at baseline after bed rest but was similar during passive standing before and after bedrest (Figure 4, middle row).

### Blood pressure and low frequency blood pressure variability

Mean arterial pressure was elevated following bedrest and in HUT, whereas there was no evidence for a group difference. Supine blood pressure variability in the low frequency range increased following 60-day of head-down tilt bed rest in the supine and in an upright position at 80° HUT (Figure 4, lower row).

### Influences of artificial gravity

We did not observe significant interactions between bedrest and the intervention for sympathetic and parasympathetic heart rate variability and baroreflex sensitivity. Tables 1–3 summarizes the group mean values on heart rate variability, blood pressure variability, and baroreflex sensitivity indices before and after bed rest for the three groups (control, continuous and intermittent centrifugation) supine and upright at 80° HUT. In addition, Table 4 summarizes the results of the mixed-effects models.

**Table 1:** Mean values and standard deviation of heart rate variability and baroreflex sensitivity parameters for the control group before and after 60-day of head-down tilt bedrest in supine and passive upright position (HUT).

	Supine before	Supine after	HUT before	HUT after
RRI (ms)	898.27 ± 138.27	701.9 ± 113.64	672.33 ± 68.82	446.31 ± 28.7
rmssd (ms)	39.81 ± 15.2	22.71 ± 14.16	18.87 ± 7.41	5.72 ± 5.58
ln rmssd (ms)	3.61 ± 0.45	2.96 ± 0.61	2.85 ± 0.49	0.97 ± 0.83
crmssd (%)	4.43 ± 1.6	3.07 ± 1.44	2.83 ± 1.09	1.25 ± 1.16
lf-RRI (ms²)	1,131.95 ± 668.05	821.2 ± 837.75	1,040.14 ± 903.55	133.66 ± 194.68
hf-RRI (ms²)	454.81 ± 304.77	173.51 ± 151.25	204.57 ± 162.03	12.07 ± 11.22
ln lf-RRI (ms²)	6.89 ± 0.6	6.31 ± 0.96	6.59 ± 1.04	3.78 ± 1.73
ln hf-RRI (ms²)	5.79 ± 1.01	4.8 ± 0.9	4.78 ± 1.38	1.86 ± 1.4
lf-RRI (nu)	72.37 ± 15.04	80.62 ± 8.18	81.22 ± 12.88	85.99 ± 6.4
hf-RRI (nu)	27.63 ± 15.04	19.38 ± 8.18	18.78 ± 12.88	14.01 ± 6.4
lf/hf-ratio (nu)	4.14 ± 3.87	5.29 ± 3.17	12.82 ± 18.6	7.99 ± 5.24
MAP (mmHg)	95.1 ± 9.62	102.67 ± 12.18	100.24 ± 14.16	112.29 ± 21.49
lf-SBP (mmHg²)	4.76 ± 3.08	9.14 ± 5.12	16.54 ± 13.09	27.41 ± 21.17
BRS-up (ms/mmHg)	19.81 ± 7.6	8.17 ± 2.87	7.17 ± 3.38	2.51 ± 0.62
BRS-down (ms/mmHg)	15.24 ± 4.01	7.74 ± 3.09	6.88 ± 3.6	1.82 ± 0.81
lf-BRS (ms/mmHg)	17.08 ± 5.98	9.55 ± 3.82	8.03 ± 2.9	1.73 ± 1

RRI, RR-Interval; Rmmsd, root mean square of successive differences; ln rmmsd, logarithmic rmssd; lf-RRI: 0.05–0.15 Hz band power of RRI variability; hf-RRI: 0.15–0.4 Hz band power of RRI variability; ln lf-RRI: logarithmic lf-RRI; ln hf-RRI: logarithmic hf-RRI; lf/hf-ratio, sympathovagal balance; MAP, mean arterial pressure; lf-SBP: 0.05–0.15 Hz band power of systolic blood pressure variability; BRS-up, Baroreflex sensitivity to an increase in systolic blood pressure; BRS-down: Baroreflex sensitivity to a decrease in systolic blood pressure; lf-BRS: baroreflex sensitivity in the low frequency band calculated by cross spectral analysis; nu, normalized units.

**Table 2:** Mean values and standard deviation of heart rate variability and baroreflex sensitivity parameters for the continuous artificial gravity group before (pre) and after (post) 60-day of head-down tilt bedrest in supine and passive upright position (HUT).

	Supine before	Supine after	HUT before	HUT after
RRI (ms)	903.69 ± 137.07	726.61 ± 120.55	676.77 ± 146.52	492.89 ± 127.13
rmssd (ms)	28.4 ± 7.4	23.37 ± 12.95	16.94 ± 10.18	10.81 ± 17.51
ln rmssd (ms)	3.32 ± 0.25	3.02 ± 0.55	2.61 ± 0.79	1.6 ± 1.24
crmssd (%)	3.15 ± 0.65	3.18 ± 1.65	2.48 ± 1.58	1.75 ± 2.32
lf-RRI (ms²)	543.75 ± 412.51	1,098.02 ± 1,216.46	548.96 ± 487.7	178.65 ± 214.07
hf-RRI (ms²)	189.68 ± 82.59	157.48 ± 101.14	101.21 ± 96.48	36.77 ± 60.21
ln lf-RRI (ms²)	6.07 ± 0.72	6.36 ± 1.25	5.9 ± 1.07	3.79 ± 2.7
ln hf-RRI (ms²)	5.13 ± 0.56	4.87 ± 0.68	4.1 ± 1.32	1.82 ± 2.65
lf-RRI (nu)	70.63 ± 11.24	77.7 ± 19.65	84.13 ± 8.58	83.03 ± 15.89
hf-RRI (nu)	29.37 ± 11.24	22.3 ± 19.65	15.87 ± 8.58	16.97 ± 15.89
lf/hf-ratio (nu)	3.03 ± 1.93	6.46 ± 4.87	7.4 ± 4.97	11.13 ± 8.33
MAP (mmHg)	95.86 ± 15.47	94.94 ± 11.33	101.82 ± 18.56	106.34 ± 17.17
lf-SBP (mmHg²)	5.74 ± 4.77	14.34 ± 10.79	21.29 ± 12.98	22.88 ± 20.72
BRS-up (ms/mmHg)	10.87 ± 5.62	8.2 ± 2.24	5.91 ± 4.59	7.2 ± 11.84
BRS-down (ms/mmHg)	13.33 ± 6.18	9.25 ± 4.04	6.28 ± 5.04	2.14 ± 0.91
lf-BRS (ms/mmHg)	12.24 ± 6.46	9.8 ± 3.61	5.2 ± 3.26	2.57 ± 2.2

RRI, RR-Interval; Rmmsd, root mean square of successive differences; ln rmmsd, logarithmic rmssd; lf-RRI: 0.05–0.15 Hz band power of RRI variability; hf-RRI: 0.15–0.4 Hz band power of RRI variability; ln lf-RRI: logarithmic lf-RRI; ln hf-RRI: logarithmic hf-RRI; lf/hf-ratio, sympathovagal balance; MAP, mean arterial pressure; lf-SBP: 0.05–0.15 Hz band power of systolic blood pressure variability; BRS-up, Baroreflex sensitivity to an increase in systolic blood pressure; BRS-down: Baroreflex sensitivity to a decrease in systolic blood pressure; lf-BRS: baroreflex sensitivity in the low frequency band calculated by cross spectral analysis; nu, normalized units.

**Table 3:** Mean values and standard deviation of heart rate variability and baroreflex sensitivity parameters for the intermittend artificial gravity group before and after 60-day of head-down tilt bedrest in supine and passive upright position (HUT).

	Supine before	Supine after	HUT before	HUT after
RRI (ms)	929.07 ± 183.18	776.8 ± 152.58	699.89 ± 148.83	487.93 ± 104.33
rmssd (ms)	31.91 ± 16.22	18.2 ± 7.98	11.79 ± 7.12	4.44 ± 3,09
ln rmssd (ms)	3.33 ± 0.59	2.81 ± 0.49	2.3 ± 0.62	1.31 ± 0.62
crmssd (%)	3.35 ± 1.55	2.28 ± 0.76	1.63 ± 0.82	0.86 ± 0.43
lf-RRI (ms²)	588.45 ± 485.4	458.47 ± 265.67	376.82 ± 330.73	100.71 ± 154.9
hf-RRI (ms²)	245.68 ± 231	85.66 ± 47.25	45.6 ± 41.70	13.35 ± 22.25
ln lf-RRI (ms²)	6.02 ± 0.95	5.96 ± 0.65	5.44 ± 1.22	3.43 ± 1.62
ln hf-RRI (ms²)	5.11 ± 0.98	4.25 ± 0.78	3.41 ± 0.99	1.33 ± 1.68
lf-RRI (nu)	69.62 ± 19.43	83.2 ± 7.63	87.2 ± 6.69	86.87 ± 8.68
hf-RRI (nu)	30.38 ± 19.43	16.8 ± 7.63	12.8 ± 6.69	13.13 ± 8.68
lf/hf-ratio (nu)	3.14 ± 1.68	6.56 ± 4.19	8.69 ± 4.78	11.19 ± 10.43
MAP (mmHg)	92.48 ± 10.14	88.46 ± 17.6	97.83 ± 15.66	96.6 ± 22.96
lf-SBP (mmHg²)	3.34 ± 2.32	9.82 ± 9.28	11.14 ± 9.97	19.94 ± 12.06
BRS-up (ms/mmHg)	17.39 ± 9.93	8.56 ± 3.77	5.50 ± 4.02	2.54 ± 2.69
BRS-down (ms/mmHg)	16.33 ± 9.08	8.55 ± 4.87	4.92 ± 3.1	2.28 ± 1.98
lf-BRS (ms/mmHg)	16.87 ± 11.25	8.84 ± 4.37	6.1 ± 3.79	1.86 ± 2.51

RRI, RR-Interval; Rmmsd, root mean square of successive differences; ln rmmsd, logarithmic rmssd; lf-RRI: 0.05–0.15 Hz band power of RRI variability; hf-RRI: 0.15–0.4 Hz band power of RRI variability; ln lf-RRI: logarithmic lf-RRI; ln hf-RRI: logarithmic hf-RRI; lf/hf-ratio, sympathovagal balance; MAP, mean arterial pressure; lf-SBP: 0.05–0.15 Hz band power of systolic blood pressure variability; BRS-up, Baroreflex sensitivity to an increase in systolic blood pressure; BRS-down: Baroreflex sensitivity to a decrease in systolic blood pressure; lf-BRS: baroreflex sensitivity in the low frequency band calculated by cross spectral analysis; nu, normalized units.

**Table 4:** Results of the linear mixed-effects models with fixed effects for timepoint, position, group, age and sex.

		Intercept	Timepoint R0 (ref. BDC-5)	Position HUT (ref. supine)	group (ref. ctrl)		Age	Sex female (ref. male)
					cAG	iAG		
RRI (ms)	Estimate [95%-CI]	787.91 [624.50–951.33]	−193.65 [−226.53–−160.76]	−245.75 [−278.63–−212.86]	36.55 [−64.81–137.90]	50.78 [−49.87–151.44]	3.9 3 [−0.61–8.46]	−84.03 [−171.38–3.32]
	*p*-value		<0.001	<0.001	0.563		0.086	0.059
Rmssd (ms)	Estimate [95%-CI]	43.96 [31.93–56.00]	−10.39 [−14.49–−6.28]	−16.0 [−20.05–−11.85]	−2.18 [−9.55–5.19]	−4.62 [−11.92–2.68]	−0.23 [−0.56–0.09]	−3.92 [−10.24–2.40]
	*p*-value		<0.001	<0.001	0.432		0.152	0.210
ln rmssd (ms)	Estimate [95%-CI]	4.23 [3.40–5.05]	−0.73 [0.99 –−0.81]	−1.07 [−1.33 – −0.91]	−0.18 [−0.69–0.33]	−0.35 [−0.85 –0.15]	−0.01 [−0.04 –0.01]	−0.24 [−0.04–0.01]
	*p*-value		<0.0001	<0.0001	0.3689		0.2473	0.2550
crmssd (%)	Estimate [95%-CI]	5.55 [4.16–6.93]	−0.90 [−1.39–−0.42]	−1.44 [−1.92–−0.95]	−0.32 [−1.17–0.52]	−0.8 [−1.64–0.04]	−0.04 [−0.08–0.00]	−0.26 [−0.99–0.47]
	*p*-value		0.0004	<0.0001	0.1583		0.0319	0.4610
lf-RRI (ms²)	Estimate [95%-CI]	1,642.12 [953.18–2,331.08]	−236.42 [−449.43–−23.4]	−373.67 [−586.69–−160.66]	−169.01 [−591.20–253.18]	−341.14 [−759.5–253.18]	−13.64 [−32.52–5.24]	−380.44 [−743.84–−17.03]
	*p*-value		0.0301	0.0008	0.2579		0.1471	0.0411
hf-RRI (ms²)	Estimate [95%-CI]	483.39 [347.15–619.63]	−127.74 [−183.55–−71.93]	−149.49 [−205.30–−93.68]	−99.00 [−181.11–−16.9]	−109.15 [−190.30–−28.00]	−3.89 [−8.94–−0.22]	−8.94 [−79.51–61.53
	*p*-value		<0.0001	<0.0001	0.0205		0.0387	0.7937
ln lf-RRI (ms²)	Estimate [95%-CI]	7.97 [6.61–9.33]	−0.11 [−0.74–0.52]	−0.35 [−0.98–0.28]	−0.35 [−1.17–0.46]	−0.57 [−1.38–0.24]	−0.03 [−0.07–0.00]	−0.65 [−1.35–0.05]
	*p*-value		<0.0001	<0.0001	0.3542		0.0683	0.0680
ln hf-RRI (ms²)	Estimate [95%-CI]	7.44 [6.13–8.76]	−0.70 [−1.32–−0.08]	−1.25 [−1.87–−0.63]	−0.44 [−1.23–0.35]	−0.73 [−1.52–0.05]	−0.05 [−0.09	0.01 [−0.67–0.69]
	*p*-value		<0.0001	0.0001	0.1679		0.0065	0.9669
lf-RRI (nu)	Estimate [95%-CI]	66.79 [54,60–78.98]	5.46 [1.36–9.57]	9.14 [5.03–13.25]	0.95 [−6.49–8.38]	2.82 [−4.55–10.19]	0.26 [−0.07–0.59]	−11.00 [−17.40–−4.60]
	*p*-value		0.0099	<0.0001	0.7209		0.1208	0.0019
hf-RRI (nu)	Estimate [95%-CI]	33.21 [21.02–45.40]	−5.46 [−9.57–−1.36]	−9.14 [−13.25–−5.03]	−0.95 [−8.38–6.49]	−2.82 [−10.19–4.55]	−0.26 [−0.59–0.07]	11.0 [4.6–17.40]
	*p*-value		0.0099	<0.0001	0.7209		0.1208	0.0019
lf/hf-ratio (nu)	Estimate [95%-CI]	1.05 [−7.43–9.53]	1.53 [−1.05–4.11]	5.06 [2.48–7.64]	0.04 [−5.16–5.24]	0.11 [−5.04–5.27]	0.12 [−0.11–0.35]	−3.07 [−7.55–1.40]
	*p*-value		0.2419	0.0002	0.9989		0.3014	0.1670
MAP (mmHg)	Estimate [95%-CI]	105.25 [83.56–126.94]	2.78 [−1.19–6.76]	7.39 [3.42–11.37]	−5.33 [−18.80–8.14]	−9.89 [−23.27–8.14]	−0.21 [−0.92–0,29]	11.44 [−0.17–23.06]
	*p*-value		0.1668	0.0004	0.3238		0.2885	0.0531
lf-SBP (mmHg²)	Estimate [95%-CI]	8.57 [−4.99–22.14]	6.83 [2.62–11.05]	12.06 [7.84–16.27]	1.3 [−7.01–9.61]	−3.41 [−11.65–4.83]	−0.11 [−0.48–0.26]	0.86 [−6.29–8.02]
	*p*-value		0.0019	<0.0001	0.4824		0.5367	0.8038
BRS-up (ms/mmHg)	Estimate [95%-CI]	20.86 [15.41–26.32]	−5.09 [−7.5–−2.09]	−7.19 [−9.59–−4.79]	−1.70 [−5.03–1.64]	−0.51 [−3.76–2.74]	−0.14 [−0.29–0.01]	−2.39 [−5.23–0.46]
	*p*-value		0.0001	<0.0001	0.5620		0.0608	0.0952
BRS-down (ms/mmHg)	Estimate [95%-CI]	20.84 [15.83–25.85]	−5.45 [−6.86–−4.03]	−7.74 [−9.14–−6.34]	0.54 [−3.65–2.57]	0.63 [−2.42–3.68]	−0.16 [−0.30–−0.03]	−3.11 [−5.79–−0.44]
	*p*-value		<0.0001	<0.0001	0.7339		0.0217	0.0250
lf-BRS (ms/mmHg)	Estimate [95%-CI]	22.61 [18.14–27.09]	−5.11 [−6.81–−3.41]	−8.06 [−9.75–−6.36]	−1.55 [−4.26–1.17]	0.05 [−2.65–2.75]	−0.18 [−0.30–−0.06]	−3.85 [−6.19–−1.51]
	*p*-value		<0.0001	<0.0001	0.3955		0.0065	0.0027

RRI, RR-Interval; Rmmsd, root mean square of successive differences; ln rmmsd, logarithmic rmssd; lf-RRI: 0.05–0.15 Hz band power of RRI variability; hf-RRI: 0.15–0.4 Hz band power of RRI variability; ln lf-RRI: logarithmic lf-RRI; ln hf-RRI: logarithmic hf-RRI; lf/hf-ratio, sympathovagal balance; MAP, mean arterial pressure; lf-SBP: 0.05–0.15 Hz band power of systolic blood pressure variability; BRS-up, Baroreflex sensitivity to an increase in systolic blood pressure; BRS-down: Baroreflex sensitivity to a decrease in systolic blood pressure; lf-BRS: baroreflex sensitivity in the low frequency band calculated by cross spectral analysis; nu, normalized units.

## Discussion

The important finding of our study is that daily 30 min artificial gravity on a short-arm centrifuge with 1Gz at the center of mass do not suffice to prevent changes in autonomic cardiovascular control following 60-day of 6° head-down tilt bed rest. Our findings are consistent with changes in autonomic control with a shift from parasympathetic to sympathetic modulation (29–32). Possibly, the response helped maintaining cardiovascular control in the face of deconditioning through simulated weightlessness. However, this adaptation may have limited the capacity of the cardiovascular system to cope with additional environmental stresses, such as standing in terrestrial gravity (33).

Head-down tilt bed rest is an established model to mimic the impact of microgravity on the human body (34). The model enables research on potential countermeasures for cardiovascular deconditioning or other weightlessness-induced health challenges before these techniques are tested or applied in space (35). Autonomic cardiovascular control is strongly affected by sodium intake and fluid levels, changed body weight, and altered circadian rhythms (36–39). The fact that all these potential confounding variables were rigorously standardized throughout the study is a particular strength. Because tight control of everyday routines and confinement to a research ward could result in psychological stress, all study participants underwent a rigorous psychological screening before inclusion (19) and trained psychologists were available for counseling throughout the study. We assessed autonomic cardiovascular control during tilt table testing before and after bed rest, to test interactions between cardiovascular deconditioning and a sufficiently strong acute environmental hemodynamic challenge on autonomic cardiovascular control (40).

Participants in the control group and in the artificial gravity groups exhibited significant heart rate increases following head down bedrest, particularly when standing. All three groups showed significant HRV reductions with a relative increase in the low frequency range following bedrest. Uncorrected and corrected rmssd and high frequency power of HRV (hf-RRI), which are established measures of parasympathetic heart rate modulation, were substantially reduced with bedrest particularly when standing (20). Low frequency power of HRV (lf-RRI), which is affected by parasympathetic and sympathetic influences on the sinus node, also decreased with bedrest albeit to a lesser degree such that the lf/hf ratio in the supine position increased (41). While the concept that the lf/hf ratio is a measure of sympathovagal balance is controversial, the findings likely suggest that autonomic heart rate control was shifted towards increased sympathetic modulation following head-down tilt bedrest (42). Baroreflex sensitivity also tended to decrease during head-down tilt bedrest, possibly less pronounced with artificial gravity. In contrast, low frequency blood pressure oscillations increased following head-down tilt bedrest in all groups. The findings further support the idea that living in microgravity be it real or simulated through head-down tilt bedrest alters autonomic cardiovascular balance towards sympathetic predominance, at least in the absence of fully effective countermeasures (43, 44).

In our study, changes in autonomic cardiovascular control towards sympathetic predominance may have contributed to an increase in mean arterial blood pressure. In contrast, a recent study employing direct sympathetic nerve recordings through microneurography showed sympathetic activation with stable blood pressure following 30-day head-down tilt bedrest (17). We dare to speculate that sympathetic activation serves as compensatory mechanism to maintain hemodynamic homeostasis and that this response may overshoot in susceptible individuals. On a shorter time scale, reflex adjustments in sympathetic activity to standing can overshoot such that blood pressure increases excessively, a phenomenon referred to as orthostatic hypertension (45). Yet, our study cannot discern whether baroreflex-mediated changes in autonomic control result from altered volume status and cardiovascular deconditioning or from direct effects of simulated weightlessness on the autonomic nervous system. The observation that autonomic dysregulation following head down tilt bed rest is attenuated with acute volume loading stresses the importance of volume status and cardiac loading conditions (46). An alternative explanation is that altered rhythmicity of sympathetic nerve discharges may change coupling between sympathetic activity and vascular responses (47, 48). Regardless of the mechanism driving the relative increase in sympathetic activity, be it secondary to reduced cardiac preload or to impaired vascular sympathetic transduction, the reserve of the autonomic nervous system to respond to additional hemodynamic stresses could be reduced. Indeed, while daily artificial gravity at least partly preserved orthostatic tolerance, time to presyncope was numerically reduced in all three groups (28).

## Limitations

The relatively low number of study participants explained by the complexity and costs of head-down tilt bed rest studies is an important limitation given the substantial interindividual variability in short-term autonomic heart rate and blood pressure control. However, our study included more participants than previous investigations (14). Moreover, we applied indirect measures of sympathetic and parasympathetic cardiovascular regulation. Invasive measurements such muscle sympathetic nerve activity may provide more direct insight on heart rate variability and autonomic activity (49). In addition, we did not measure respiratory rate or volume, which could confound our results. Finally, newer methods of time series analysis being able to analyze shorter periods especially during standing might provide deeper insight, like cross wavelet analysis (50). Possibly, longer artificial gravity duration or intensity are required to achieve advantages in autonomic cardiovascular control by further prevention of cardiovascular deconditioning.

## Conclusion and outlook

In the absence of sufficient countermeasures, the extreme environmental conditions in space and simulated weightlessness through head-down tilt bed rest decrease the ability of the cardiovascular system to cope with acute hemodynamic challenges. In our study, daily artificial gravity up to 30 min a day elicited through short arm centrifugation did not prevent changes in autonomic cardiovascular control during 60-day of head-down tilt bed rest. Moreover, while artificial gravity attenuated reductions in orthostatic tolerance with head-down tilt bed rest, orthostatic tolerance was not fully maintained (28). Perhaps, artificial gravity dosing has to be increased or combination with other countermeasures is required to increase efficacy in maintaining autonomic cardiovascular control. Alternative approaches simulating orthostatic challenges in weightlessness, such as lower body negative pressure (51), are explored in current head-down tilt bed rest studies. Severe orthostatic intolerance was common during early space missions, however, severely symptomatic orthostatic intolerance seems to be uncommon nowadays even after prolonged space missions (52). Given the changing face of human spaceflight in the near future, the question whether autonomic cardiovascular control is sufficiently preserved with current countermeasures should be scrutinized. On missions to the Moon or to Mars, human beings will be exposed to orthostatic stress on another celestial body following deconditioning in space. While gravitational stress is lower than on Earth, the cardiovascular system will also be burdened by psychological and physiological stresses imposed by altered circadian rhythms, suboptimal nutrition, and frequent extravehicular activities (34). Reductions in orthostatic tolerance or physical performance may not be acceptable. According to recently published reference values for RRI-corrected rmssd, changes in this measurement in our study correspond to those observed with ten years aging in men and 15 years aging in women (53). Fortunately, changes in autonomic cardiovascular control after bedrest are rapidly reversible within days following reconditioning (54). Finally, our study suggests that non-invasive cardiovascular autonomic measurements, which can be applied in space using lightweight and easy-to-use wearables, are remarkably sensitive in detecting changes in autonomic control (55). We propose that such measurements could have utility in individualizing countermeasure application and in planning stressful activities such as extravehicular activities, which may have to be avoided when cardiovascular autonomic control is challenged even at rest.

## Data Availability

The original contributions presented in the study are included in the article/Supplementary Material, further inquiries can be directed to the corresponding author.
